# Vitamin D Induces Interleukin-1β Expression: Paracrine Macrophage Epithelial Signaling Controls *M. tuberculosis* Infection

**DOI:** 10.1371/journal.ppat.1003407

**Published:** 2013-06-06

**Authors:** Mark Verway, Manuella Bouttier, Tian-Tian Wang, Marilyn Carrier, Mario Calderon, Beum-Soo An, Emmanuelle Devemy, Fiona McIntosh, Maziar Divangahi, Marcel A. Behr, John H. White

**Affiliations:** 1 Department of Physiology, McGill University, Montreal, Quebec, Canada; 2 Meakins-Christie Laboratories, McGill University, Montreal, Quebec, Canada; 3 Montreal General Hospital, McGill University, Montreal, Quebec, Canada; 4 Department of Medicine, McGill University, Montreal, Quebec, Canada; 5 Division of Infectious Diseases and Medical Microbiology, McGill University, Montreal, Quebec, Canada; University of Massachusetts, United States of America

## Abstract

Although vitamin D deficiency is a common feature among patients presenting with active tuberculosis, the full scope of vitamin D action during *Mycobacterium tuberculosis* (*Mtb*) infection is poorly understood. As macrophages are the primary site of *Mtb* infection and are sites of vitamin D signaling, we have used these cells to understand the molecular mechanisms underlying modulation of the immune response by the hormonal form of vitamin D, 1,25-dihydroxyvitamin D (1,25D). We found that the virulent *Mtb* strain H37Rv elicits a broad host transcriptional response. Transcriptome profiling also revealed that the profile of target genes regulated by 1,25D is substantially altered by infection, and that 1,25D generally boosts infection-stimulated cytokine/chemokine responses. We further focused on the role of 1,25D- and infection-induced interleukin 1β (IL-1β) expression in response to infection. 1,25D enhanced IL-1β expression via a direct transcriptional mechanism. Secretion of IL-1β from infected cells required the NLRP3/caspase-1 inflammasome. The impact of IL-1β production was investigated in a novel model wherein infected macrophages were co-cultured with primary human small airway epithelial cells. Co-culture significantly prolonged survival of infected macrophages, and 1,25D/infection-induced IL-1β secretion from macrophages reduced mycobacterial burden by stimulating the anti-mycobacterial capacity of co-cultured lung epithelial cells. These effects were independent of 1,25D-stimulated autophagy in macrophages but dependent upon epithelial IL1R1 signaling and IL-1β-driven epithelial production of the antimicrobial peptide DEFB4/HBD2. These data provide evidence that the anti-microbial actions of vitamin D extend beyond the macrophage by modulating paracrine signaling, reinforcing its role in innate immune regulation in humans.

## Author Summary

In 2010 there were ∼9 million cases of tuberculosis and 1.4 million deaths, representing the second largest cause of death worldwide and the leading cause of death from a curable disease. *M. tuberculosis* (*Mtb*) replicates within cells of the immune system called macrophages over an approximate 72 hour period, ultimately inducing cell death. Notably, macrophages are sites of vitamin D signaling, and there is broad evidence that vitamin D modulates macrophage responses to *Mtb*. Elevated levels of TB have long been associated with vitamin D deficiency, strongly suggesting that vitamin D supplementation may be of therapeutic benefit. In this study we profile the host macrophage response to *Mtb* infection through the use of high-throughput genomics techniques. From this we have discovered that the dominant function of vitamin D is the modulation of the levels of specific cytokines, mediators of immune cell to cell signaling. Of particular interest was the increase in IL-1β signaling, which we show to be directly regulated by vitamin D. We also show that this increase in IL-1β is critical for driving a signaling cascade between macrophages and lung epithelial cells leading to epithelial antimicrobial peptide production that helps to contain *Mtb* infection in our model culture system.

## Introduction

*Mycobacterium tuberculosis* (*Mtb*) infects ∼2 billion people [1] and active tuberculosis (TB) represents the leading cause of death from a curable disease [2]. The typical *Mtb* life-cycle involves entry into the host via inhalation (exposure), survival and replication of bacteria in the lungs and associated lymph nodes where they resist host elimination (infection), and ultimately promotion of host immunopathology resulting in aerosol transmission via coughing to the next host (disease). While current treatments aim to control active disease or prevent progression from infection to disease, an attractive point of intervention would be at the transition from exposure to infection by enhancing innate pathogen control at the time of exposure.

Innate immunity to *Mtb* infection is critical for determining disease outcome, and it has long been recognized that during TB outbreak investigations, only 30–50% of those with an equivalent exposure develop a productive infection, as demonstrated by a tuberculin skin-test conversion [3]. The primary site of *Mtb* infection is alveolar macrophages, which lie within the alveolar space, adjacent to the epithelial lining [4]. Lung epithelial cells are generally not considered part of the innate immune responses to *Mtb* infection. However, they represent both a physical and an immunological barrier to infection by contributing to the maintenance of mucosal integrity, promoting phagocytosis, and producing several antimicrobial peptides, vanguards of innate immune responses to infection [4], [5]. For example, antimicrobial peptide (AMP) DEFB4/HBD2 is expressed in upper airway epithelial cells, is inducible by interleukin-1β (IL-1β), and has been detected in bronchoalveolar lavage fluid from normal healthy humans [6]. It has been shown to have direct antimicrobial activity against *Mtb* and drug-resistant *Mtb* [7].

IL-1β is critical for host resistance to *Mtb* infection, demonstrated by the substantially reduced survival of IL-1β−/− or IL1R−/− mice after infection [8], [9], [10], [11]. Caspase-1 cleaves a precursor of IL-1β to generate its active form. Catalytically active caspase-1 binds to the apoptosis-associated speck-like protein containing a caspase recruitment domain (ASC) subunit of inflammasomes, multiprotein complexes containing pattern recognition receptors (PRRs), which detect infection by pathogens or cellular stress. A number of PRRs have been implicated in the detection of intracellular pathogens, and the resulting IL-1β secretory response. For example, AIM2 is a cytoplasmic sensor for double stranded DNA (dsDNA) and has recently been implicated as a component of the caspase-1 inflammasome in cells infected with viral and intracellular bacterial pathogens [12], [13], [14], [15], [16]. Similarly, NOD2 is a cytoplasmic sensor for muramyl dipeptide [17], which stimulates NF-κB signaling to induce IL-1β expression [18], plays an important role in immunity against *Mtb* [19], and associates with the NALP1-containing inflammasome [20]. Lastly, the NLRP3 pattern recognition receptor is activated by a wide range of signals [21]. It is of particular importance for IL-1β secretion from macrophages after infection with *Mycobacteria marinum* (*M. marinum*) [22] and *Mtb* [23].

Given the critical role of innate immunity in initiating an effective response to *Mtb* infection, we determined in detail the host macrophage transcriptomic and cytokine responses to virulent H37Rv infection and modulation of this response by the hormonal form of vitamin D, 1,25-dihydroxyvitamin D (1,25D). Historically, there is a correlation between vitamin D deficiency and TB susceptibility [24], [25]. Epidemiologic studies have documented a higher occurrence of active TB during winter months when sunlight exposure is reduced [26] and vitamin D deficiency has been identified as a common feature of patients with active TB [27], [28], [29]. However, the mechanisms underlying vitamin D signaling and control of *Mtb* infection are not well understood. 1,25D synthesis is induced in macrophages and dendritic cells upon exposure to pathogen, and the 1,25D-bound vitamin D receptor (VDR) directly induces transcription of genes encoding AMPs DEFB4/HBD2 and cathelicidin antimicrobial peptide (CAMP) [30], [31], [32], [33], [34], [35], both of which have demonstrated anti-mycobacterial activity [36], [37]. Despite this, the direct effects of 1,25D on *Mtb* bacterial burden in infected macrophages have been modest [38], [39], posing the question of whether these findings alone could account for the clinical and epidemiological observations, if they are indeed causally associated.

Here, we show that one of the primary macrophage responses to *Mtb* infection is a broad cytokine/chemokine response, which is generally enhanced by 1,25D. Importantly, 1,25D directly stimulates *IL1B* gene transcription, a critical component of the macrophage response to *Mtb* infection [40]. As this mechanism of *IL1B* regulation was not conserved in mice, we developed a co-culture system between macrophages and primary small airway lung epithelial cells to model the effects of elevated IL-1β secretion. In these co-culture experiments, 1,25D potentiated IL-1β signaling from macrophages resulting in the secretion of DEFB4 from primary lung epithelial cells. Taken together these results suggest that the effects of 1,25D extend beyond the macrophage and involve the modulation of paracrine signaling to enhance the innate immune responses to *Mtb* infection.

## Results

### 1,25D enhances expression and secretion of cytokines and chemokines in *Mtb*-infected macrophages

**Figure 1:**
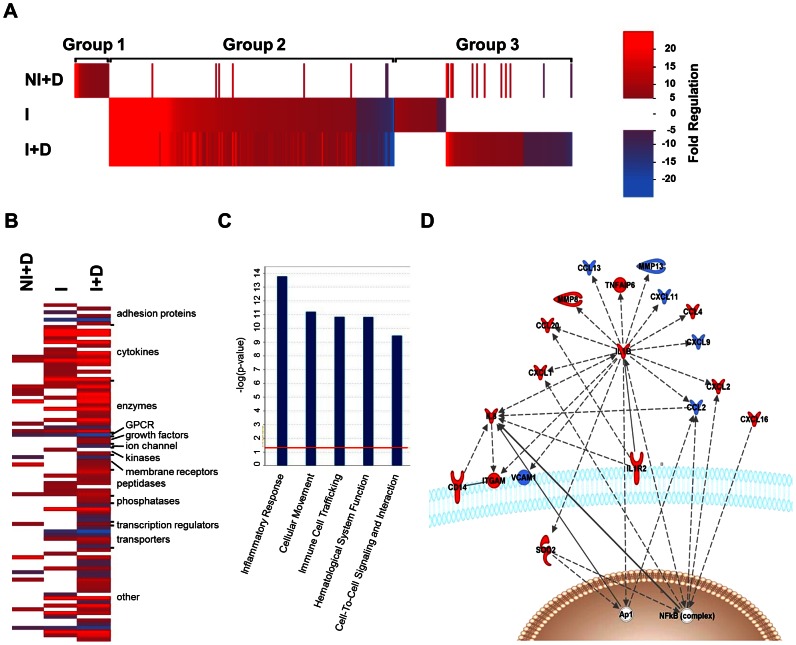
1,25D alters the host macrophage transcriptomic response to *Mtb* infection. (**A**) Intensity heat map of genes regulated 5-fold or more during infection in the absence or presence of 1,25D. THP-1 cells were either not infected (NI) or infected with H37Rv (I) and treated with vehicle (DMSO) or 100 nM 1,25D (+D) for 24 hours. Each vertical line represents one gene that was either up-regulated (red), down-regulated (blue) or not affected (white) under each of the conditions relative to uninfected cells not treated with 1,25D, as indicated in the scale. Group 1 represents those genes that were only detected in the NI+D condition, group 2 were those genes that were commonly expressed in both infected conditions, and group 3 represents those genes that were expressed in only one of the infected conditions as a result of 1,25D treatment. (**B**) Functional clustering heat map of genes selected for a >5-fold change in either the I or I+D condition relative to the NI control as well as having a >1.5-fold difference between the two. Increasing brightness for red and blue denote up- and down-regulation respectively. (**C**) Highest-rated functions associated with relative expression of I+D/I in genes from **B**, with a Fisher's exact test p-value threshold set at 0.01 (red line) using Ingenuity Pathways Analysis (IPA) software. (**D**) Network clustering analysis of genes from **B** using IPA software. Solid and hashed lines denote known direct and indirect actions between two proteins as determined by IPA. Red and blue denote relative up- or down-regulation of their expression in the I+D condition as compared to the I condition, respectively. Data is derived from analysis of Affymetrix Human Gene 1.0ST microarray chips. mRNA samples for analysis were prepared in triplicate, and data presented is representative of two independent replicates.

In order to understand the host macrophage transcriptional response to *Mtb* infection, we performed expression profiling studies in PMA-differentiated human THP-1 macrophage cells. Cells were infected with virulent *Mtb* strain H37Rv (I) or left uninfected (NI), and treated with vehicle (DMSO) or 100 nM 1,25D (+D) for 24 hours. 1,25D treatment of *Mtb*-infected macrophages produced broad changes in mRNA profiles (**Table S1**), in which expression of 328 genes was altered at least 5-fold by either infection or 1,25D (**Figure 1A** **, Table S2**). A heat map of highly induced transcripts identified three major groups of genes: those that were regulated by 1,25D in uninfected cells, but not in infected cells (group 1), those that were regulated in the same direction in infected cells treated with vehicle or 1,25D (group 2), and those that were regulated in infected cells in either the vehicle or 1,25D treated condition, but not both (group 3). From this it is clear that about half of all 1,25D target genes in uninfected macrophages (NI+D) are not regulated in infected cells, as they do not belong to group 1. Infection resulted in broad changes in transcription, which substantially changed the cohort of genes regulated by 1,25D, indicated by group 3, and the columns that change in intensity in group 2 (**Figure 1A**).

To understand the dominant effects of 1,25D in *Mtb*-infected macrophages, the 328 genes regulated more than 5-fold by infection were filtered to retain those whose expression was altered at least a further 1.5-fold by 1,25D (**Figure 1B**). Functional clustering of the resulting 94 genes revealed that the largest classification of these encoded cytokines (**Fig. 1B** **)**, and Ingenuity IPA network clustering strongly implicated a role in inflammatory responses, immune cell trafficking, and signaling (**Figure 1C**). IPA Pathway clustering of these revealed the strongest effects were on secreted factors, many of which were further induced by 1,25D (**Figure 1D**). To confirm the transcriptional changes at the translational levels, the supernatants from the cells used for microarray were subjected to Milliplex Human Cytokine Assay for a wide range of cytokines and chemokines analysis. We found that, in agreement with microarray data, 1,25D significantly enhanced secretion of IL-1β, CCL3/MIP1α, CCL4, CCL8, TNFα, IL-8/CXCL8, and CCL20 from macrophages infected with *Mtb* (**Table S3**).

### Production of mature 1L-1β requires caspase-1 and NLRP3 in *Mtb* infection

**Figure 2:**
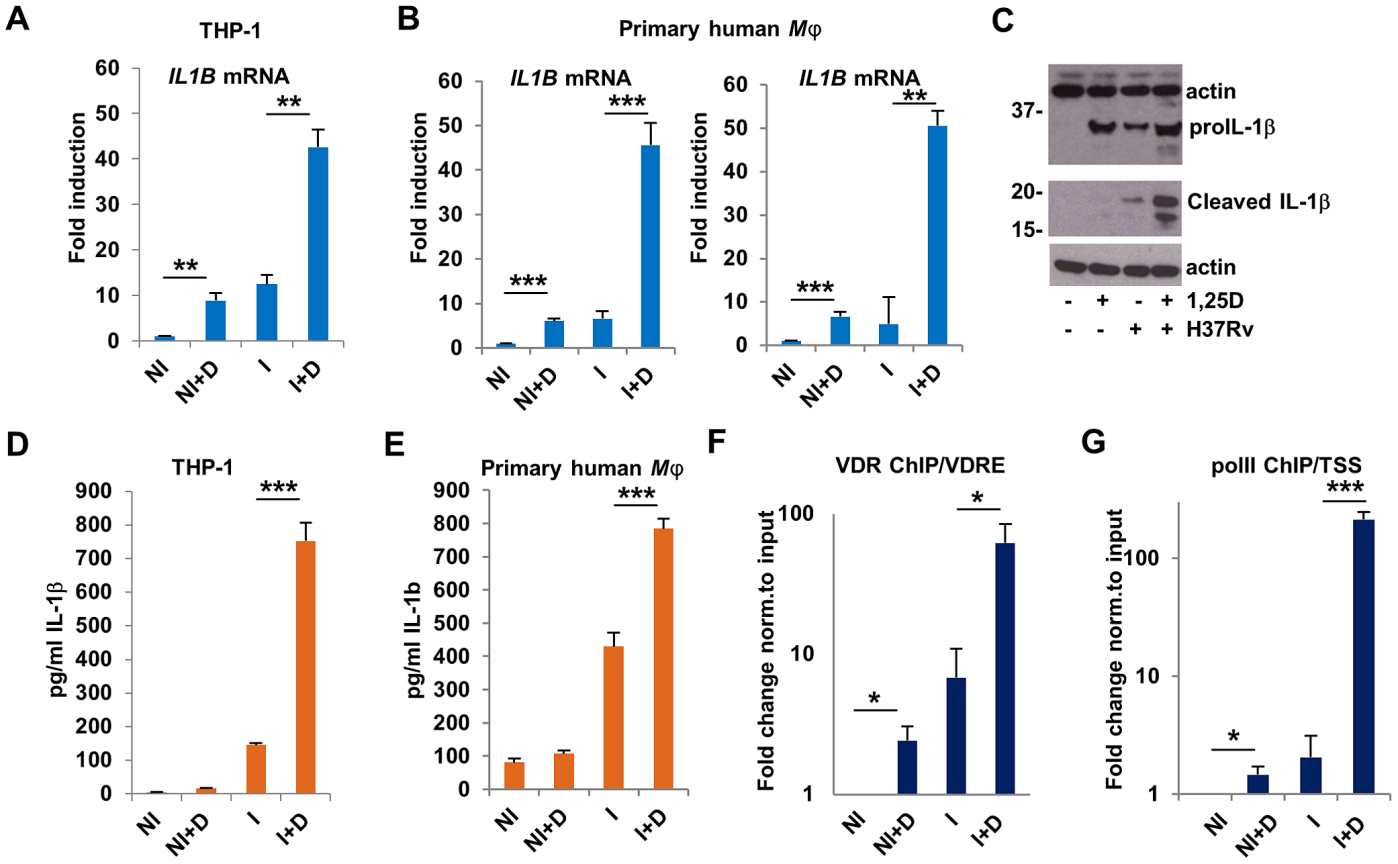
1,25D directly regulates IL-1β expression in *Mtb*-infected macrophages. (**A**) Expression of *IL1B* transcripts as assessed by RT/qPCR in control or H37Rv-infected THP-1 cells 24 hours after infection. Data are normalized to the uninfected, untreated control (NI, uninfected cells; NI+D, uninfected cells treated with 1,25D; I, H37Rv-infected cells; I+D, infected cells treated with 1,25D). (**B**) Expression of *IL1B* transcripts in two independent cultures of H37Rv-infected primary human macrophages, analyzed as in A. (**C**) Protein expression and processing of IL-1β in uninfected and infected THP-1 cells. (**D**) Secretion of IL-1β from uninfected and infected THP-1 cells. (**E**) Secretion of IL-1β from uninfected and infected primary human macrophages. (**F**) ChIP analysis of association of the VDR with the *IL1B* VDRE and transcription start site (TSS). (**G**) ChIP analysis of recruitment of the large subunit of RNA PolII to the VDRE and TSS of the *IL1B* gene. ChIP values are normalized to input for each condition and expressed as a fold relative to non-specific IgG control. All data are from one experiment and representative of at least three independent experiments (n = 3, mean, s.d.). *P<0.05, **P<0.01, ***P<0.001 as determined by Student's t-test.

Considering the critical role of IL-1β in immunity to *Mtb* infection [8], [9], [41], we next investigated the mechanisms by which 1,25D increased the expression and secretion of IL-1β in *Mtb*-infected macrophages. Consistent with the microarray data, RT/qPCR analysis showed that 1,25D up-regulated the expression of *IL1B* transcripts in uninfected and *Mtb*-infected macrophages (**Figure 2A**). Essentially identical results were obtained with two independent cultures of primary human macrophages (**Figure 2B**). Although the levels of pro-IL-1β were elevated in both 1,25D-treated macrophages as well as *Mtb*-infected macrophages treated with 1,25D, mature IL-1β was only detected in extracts of *Mtb*-infected cells treated with 1,25D (**Figure 2C**). Results of western blotting were consistent with analysis of IL-1β released from THP-1 cells or primary human macrophages, where secretion was observed only in supernatants of infected cells, and significantly elevated upon treatment with 1,25D (**Figures 2D, E**).

Previous *in silico* studies [42] identified a promoter-proximal sequence corresponding to a consensus vitamin D response element (VDRE) in the *IL1B* gene (**Fig. S1**). To further investigate the mechanisms of *IL1B* gene expression by 1,25D, we evaluated the VDR binding to this element by chromatin immunoprecipitation (ChIP) assay. VDR binding was 1,25D-dependent, and significantly enhanced by infection (**Figure 2F**). 1,25D-dependent binding of the VDR may be due in part to its elevated expression, which was stimulated by 1,25D (**Figure S2**), consistent with the *VDR* being a 1,25D target gene [43]. A similar profile was observed with RNA polymerase II (polII) binding at the *IL1B* transcription start site (TSS) (**Figure 2G**). Collectively, these experiments show the 1,25D-bound VDR stimulates transcription of the *IL1B* gene. To determine the extent of conservation of this mechanism, we performed a comparison of the *IL1B* VDRE loci across mammalian species using the UCSC genome browser [44]. The VDRE and the surrounding regions are well conserved in the genomes of non-human primates, but not in the mouse, rabbit, or guinea pig (**Figure S3**), which are commonly used to model *Mtb* infection *in vivo* [45]. Indeed, while 1,25D significantly enhanced *cyp24* expression in mouse macrophages, no regulation of *il1b* was seen (**Figure S4**), in contrast to the 6–8-fold induction of *IL1B* expression seen in uninfected THP-1 cells or primary human macrophages (**Figures 2A, B**) indicating that mice would not serve as a viable *in vivo* model to understand this effect.

**Figure 3:**
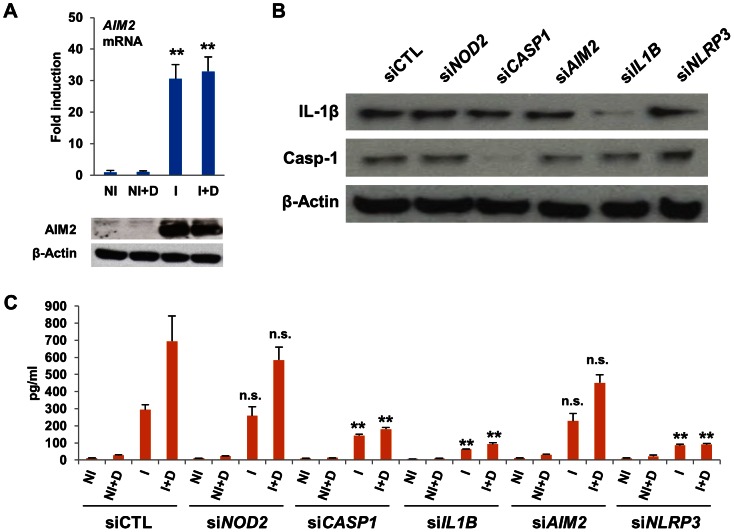
1,25D-induced IL-1β secretion requires NLRP3. (**A**) Expression of AIM2 transcripts and protein in control and infected cells 24 hours after infection. (NI, uninfected cells; NI+D, uninfected cells treated with 1,25D; I, H37Rv-infected cells; I+D, infected cells treated with 1,25D). Values are expressed as fold relative to NI. (**B**) Western blots of pro-IL-1β, caspase-1, and β-actin from THP-1 cells transfected with siRNAs indicated and infected for 24 hours with H37Rv. **C.** IL-1β secretion from uninfected (NI) or infected (I) THP-1 cells transfected with siRNAs indicated. 1,25D (D) was added as indicated. All data are from one experiment and representative of at least three independent experiments (n = 3, mean, s.d.). **P<0.01 as determined by Student's t-test relative uninfected (A) or respective siCTL (C) control.

Pro-IL-1β can be cleaved into its mature form by caspase-1 [46]. Western blots of lysates from infected macrophages revealed that neither 1,25D nor infection altered expression of caspase-1 in its pro-form (**Figure S5A**). Caspase-1 enzymatic activity was significantly higher in cytosolic lysates from *Mtb*-infected cells treated with 1,25D (**Figure S5B**). Catalytically active caspase-1 is a component of inflammasomes, of which ASC is a core component. We found that while the 23 kD form of ASC was predominant in THP-1 cells, the 20 kD splice variant [47], [48] coimmunoprecipitated with caspase-1, an association only seen in infected cells and found to be slightly higher in infected cells treated with 1,25D, consistent with elevated cytosolic caspase-1 activity under these conditions (**Figure S5C**). Inflammasomes also contain pattern recognition receptors (PRR) that detect infection or cellular stress [46], [49]. To understand which inflammasome PRR, or combination of PRRs, was responsible for the 1,25D-driven secretion of IL-1β, we investigated the potential roles of NOD2, NLRP3, and AIM2 in regulating IL-1β maturation in infected cells. Each of these has been previously implicated in detection of *Mtb* or other intracellular pathogens and in stimulating inflammasome-mediated IL-1β maturation [22], [50], [51]. Notably, expression of AIM2, a cytoplasmic sensor for dsDNA [13], [15], was induced ∼30-fold in *Mtb*-infected macrophages by RT/qPCR and western blotting (**Figure 3A**), suggesting that the AIM2 inflammasome may contribute to IL-1β cleavage and secretion.

Expression of *AIM2*, *NOD2* or *NLRP3* was strongly reduced by siRNA-mediated knockdown (**Figure S6**), along with positive controls *IL1B* and *CASP1*, in *Mtb*-infected macrophages. None of the knockdowns of PRRs had any effect on expression of pro-IL-1β protein or on expression of caspase-1 (**Figure 3B**). Depletion of NOD2 or AIM2 expression had no significant effect on IL-1β secretion (**Figure 3C**). In contrast, knockdown of NLRP3 essentially abolished IL-1β secretion from *Mtb*-infected macrophages (**Figure 3C**). These findings suggest that induced AIM2 expression is not primarily responsible for driving IL-1β maturation, but are consistent with other studies showing that IL-1β production during *Mtb* infection is largely controlled by NLRP3 [22], [23]. In contrast, they are not consistent with the finding that NOD2 function is of primary importance [51].

### 1,25D enhances IL-1β secretion from *Mtb*-infected macrophages, inducing *DEFB4* gene expression in primary lung epithelial cells

**Figure 4:**
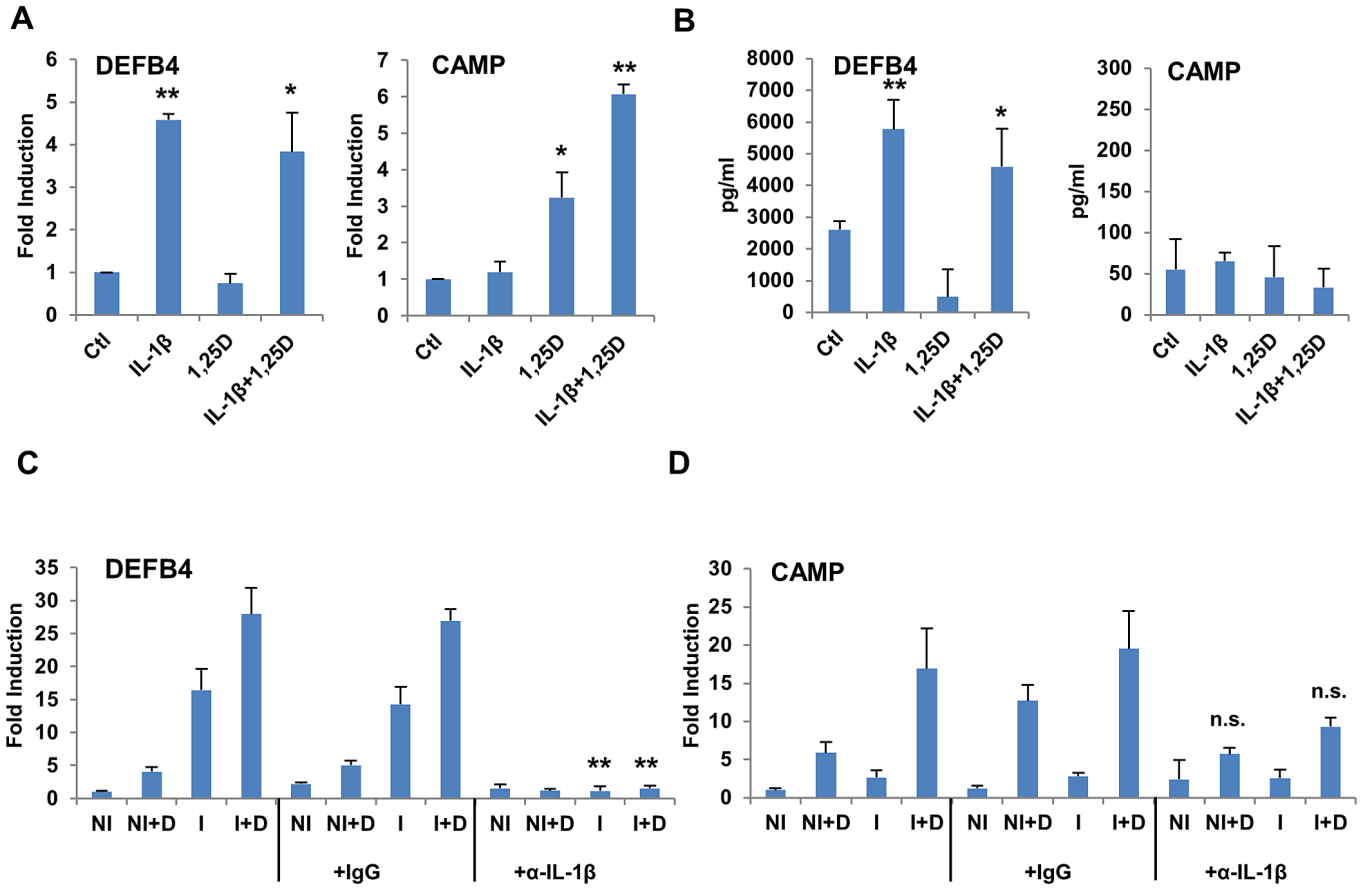
*DEFB4* and *CAMP* genes are regulated by IL-1β and 1,25D in SAECs. (**A**) Expression of *DEFB4* and *CAMP* in SAECs as measured by RT/qPCR. Cells were incubated with IL-1β (10 ng/ml) or 1,25D (100 nM) for 24 h. (**B**) Media supernatants from cells in (A) were tested for DEFB4 and LL-37 protein secretion by ELISA. (**C**) **and** (**D**) Expression of *DEFB4* (C) and *CAMP* (D) genes in SAECs incubated with conditioned media from uninfected (NI) or H37Rv-infected (I) THP-1 cells treated with vehicle or 100 nM 1,25D (+D) for 24 hours. Media was treated with neutralizing antibody against IL-1β (α-IL-1β), or normal serum IgG, as indicated, for 30 minutes prior to incubation with cells. Values are expressed as a fold of the NI control. All data are from one experiment and representative of three independent experiments using separate donors of SAECs (n = 3, mean, s.d.). *P<0.05, **P<0.01 as determined by Student's t-test relative untreated (A, B) or respective IgG (C) control.

Upon infection, alveolar macrophages initially phagocytose *Mtb*. Importantly, alveolar macrophages are also in direct contact with the respiratory epithelial surface [4]. Although the immunological contribution of lung epithelia is well studied in other contexts [52], it is poorly characterized during the course of *Mtb* infection. IL-1β induces expression of genes encoding AMPs in epithelial cells through its capacity to stimulate the activity of transcription factor, NF-κB, leading to secretion of antimicrobial proteins [53], [54]. Thus, to understand the potential crosstalk between alveolar macrophages and the human respiratory epithelial surface, we conducted a series of experiments using an *in vitro* co-culture system between macrophages and human primary cultures of non-polarized small airway epithelial cells (SAECs) from multiple donors. In cultures of SAECs alone, recombinant IL-1β induced expression of the gene encoding DEFB4, while 1,25D had no significant effect (**Figure 4A**). Conversely, induction of *CAMP* gene expression was largely 1,25D-dependent (**Figure 4A**). Media supernatants from these treated SAECs were assayed for DEFB4 and CAMP secretion by ELISA. DEFB4 was detected under control conditions, and was found to be elevated when SAECs were treated with rIL-1β, but not 1,25D (**Figure 4B**). In contrast, the cleaved C-terminal of CAMP, LL-37, was not detected above background signal under any of the conditions (**Figure 4B**). In comparison, neither DEFB4 nor LL-37 was detected in media supernatants from THP-1 cells infected with *Mtb* using this assay (data not shown).

SAECs were then cultured with conditioned media from control or H37Rv-infected macrophages to test for effects of 1,25D and secreted factors on AMP expression. In addition, as IL-1β has been shown to induce NF-κB signaling through IL1R1 [54], we investigated the expression of NF-κB target gene *DEFB4* [53], [54] in lung epithelial cells. Media from infected macrophages induced expression of *DEFB4* (**Figure 4C**) in SAECs. Importantly, pre-incubation of the transferred media with IL-1β-neutralizing antibody abolished *DEFB4* gene induction. Given that *DEFB4* expression levels are driven by IL-1β, the combined effects of 1,25D and infection on *DEFB4* transcription are consistent with levels of IL-1β secretion induced in 1,25D-treated, infected macrophages (**Figure 2D,E**). As expected, *CAMP* expression in SAECs was dependent on 1,25D (**Figure 4D**).

### Co-culture of macrophages and SAECs enhances macrophage survival and helps control *Mtb* infection

**Figure 5:**
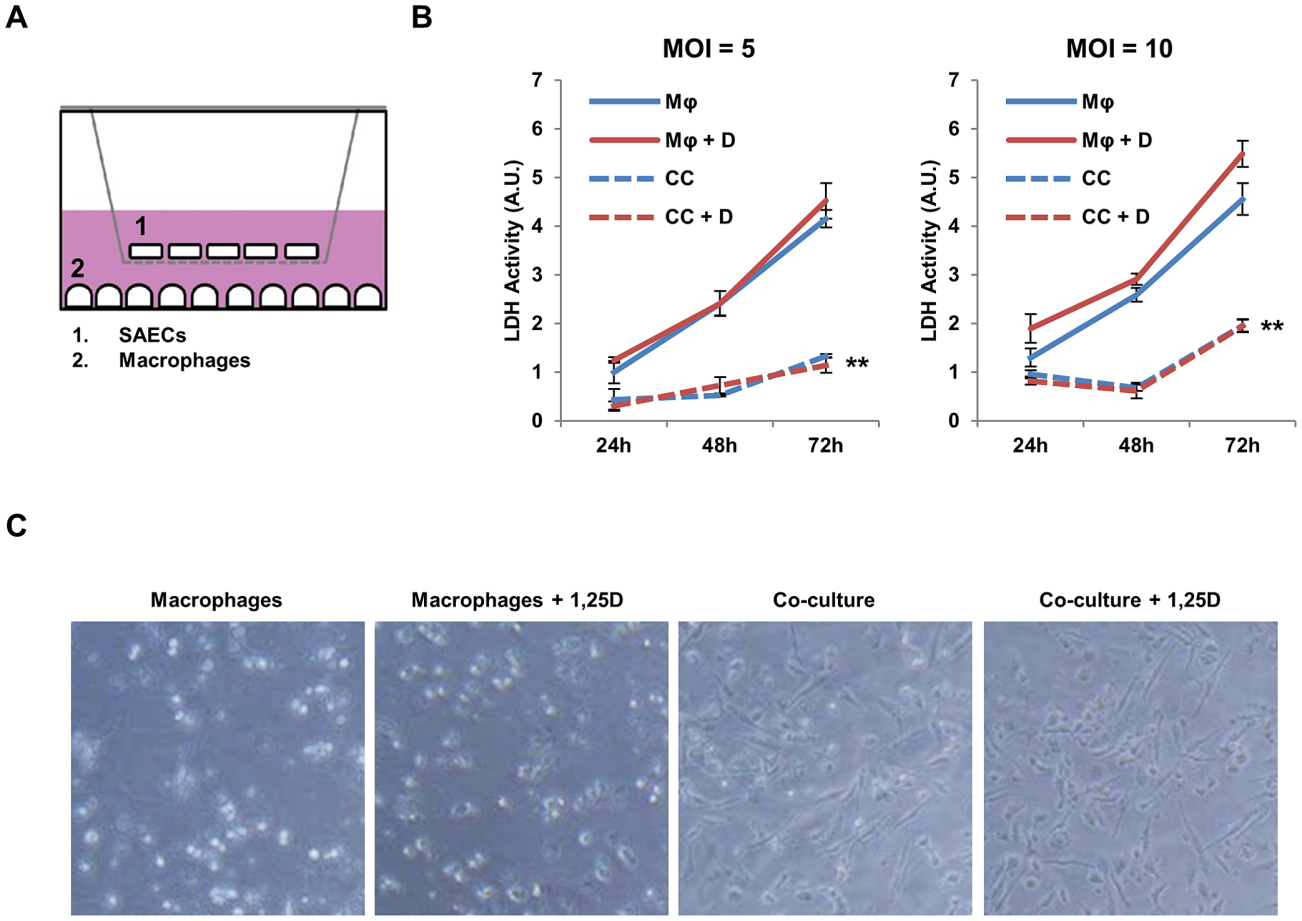
Co-culture with SAECs enhances survival of *Mtb*-infected macrophages. (**A**) Schematic representation in profile of co-culture system. THP-1 cells were cultured and infected in the lower well and SAECs were seeded in the upper transwell bucket. (**B**) LDH activity measured in the media supernatant from macrophages (Mφ) infected with H37Rv at an MOI of 5 or 10, as indicated, and treated with vehicle control or 100 nM 1,25D (+D), with and without the presence of SAECs in transwell co-culture (CC). Signal was normalized to spontaneous LDH release levels from uninfected macrophages, as measured from media supernatant collected from cells at each day. All data is expressed relative to LDH activity of the Mφ condition infected at an MOI of 5 at 24 hours post-infection. Data are from one experiment and representative of two independent experiments using separate donors of SAECs (n = 3, mean, s.d.). **P<0.01 as determined by two-way ANOVA comparing Mφ to CC conditions. (**C**) Representative phase-contrast microscopy of macrophages 3 days after infection with and without 1,25D treatment in the presence or absence of SAECs in co-culture. In the macrophage only condition, most of the cells visible appear round and out of focus as they are floating freely in the media, while cells co-cultured with SAECs are almost all adherent and exhibit normal macrophage morphology.

Next, to model the consequences of the real-time interaction between infected macrophages and the alveolar epithelia during the initial round of infection, we established a transwell co-culture system between *Mtb*-infected macrophages and SAECs (**Figure 5A**). Macrophages were infected with *Mtb* for 4 hours and washed extensively to eliminate any remaining extracellular mycobacteria. A transwell bucket containing co-cultured lung epithelial cells was then added to the tissue culture plate containing the infected macrophages. The two cell populations were separated by a 0.4 µm filter to allow for the exchange of secreted proteins but prevent migration of mycobacteria. Note that in control experiments no mycobacteria were detected in the transwell bucket 4 days after infection (data not shown). Co-culture of macrophages infected at an multiplicity of infection (MOI) of 5 with SAECs dramatically extended macrophage cell survival at three days post-infection, as measured by cytoplasmic lactate dehydrogenase (LDH) release (**Figure 5B**), a marker of plasma-membrane compromise and necrosis. After 3 days of infection, the relative amount of LDH release was comparable to that seen 24 hours after infection under macrophage-only conditions. 1,25D treatment had no effect on the survival of infected cells cultured in the absence or presence of SAECs. When macrophages infected at an MOI of 10 were co-cultured with SAECs, LDH release was slightly higher, but a similar protective effect of SAECs was observed (**Figure 5B**). The protective benefit of the SAECs in co-culture was also clear by the relative amount of adherent cells remaining as visualized by phase-contrast microscopy (**Figure 5C**); most of the macrophages co-cultured with SAECs were still adherent, whereas infected macrophages cultured in the absence of SAECs had detached from the plate.

**Figure 6:**
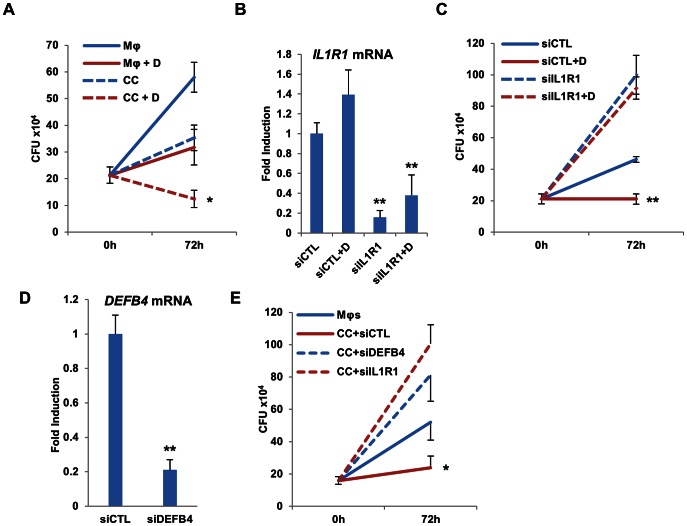
Control of *Mtb* infection by co-cultured epithelial cells is dependent on epithelial IL1R1 and DEFB4 expression. (**A**) CFU of macrophages (Mφ) infected with H37Rv at an MOI of 5 for 4 hours and treated with vehicle control or 100 nM 1,25D (+D), with and without the additional presence of SAECs in transwell co-culture (CC). Data are from three experimental replicates (mean and SD) and representative of three independent experiments using different donors of primary cells. Statistical significance was determined by one-way ANOVA. (*P<0.05). (**B**) Validation of siRNA-mediated knockdown of *IL1R1* expression in SAECs. RNA was extracted from SAECs cells 3 days after the initiation of co-culture and 4.5 days after transfection of control siRNA (siCTL) or siIL1R1. Data are from three experimental replicates (mean and SD), **P<0.01 as determined by student's T-test relative to respective siCTL controls. (**C**) CFU quantification of *Mtb* in THP-1 cells infected at an MOI of 5 for 4 hours after 72 hours of co-culture with SAEC cells transfected with control siRNA (siCTL) or siRNA specific to *IL1R1*. 1,25D (D) was added as indicated. Data are from three experimental replicates (mean and SD) and representative of two independent experiments. Statistical significance was determined by one-way ANOVA. (**P<0.01). (**D**) Validation of siRNA-mediated knockdown of *DEFB4* expression in SAECs. RNA was extracted from SAECs cells 3 days after the initiation of co-culture and 4.5 days after transfection of control siRNA (siCTL) or siDEFB4. Data are from three experimental replicates (mean and SD) and representative to two independent replicates. **P<0.01 as determined by student's T-test relative to siCTL control. (**E**) CFU quantification of *Mtb* in THP-1 cells infected at an MOI of 5 for 4 hours after 72 hours of co-culture with SAEC cells transfected with control siRNA (siCTL) or siRNA specific to *IL1R1* or *DEFB4*. Data are from three experimental replicates (mean and SD) and representative of two independent experiments using separate donors of SAECs. Statistical significance was determined by one-way ANOVA. (*P<0.05).

To determine any effects of co-culturing with and without 1,25D on mycobacterial burden, colony forming unit (CFU) assays were performed with cells co-cultured as above. We determined changes in total *Mtb* burden after 72 hours of infection. The addition of SAECs resulted in a halving of mycobacterial burden at this time point, and addition of 1,25D to the co-culture system produced a further significant reduction in mycobacteria (**Figure 6A**). To confirm the contribution of epithelial signaling by infection- and 1,25D-induced IL-1β towards the reduction in mycobacterial burden, we knocked down IL1R1 receptor expression in SAECs 36 hours prior to their co-culture. Control experiments showed that expression of epithelial IL1R1 was reduced for at least 72 hours after siRNA-mediated knockdown (**Figure 6B**). Mycobacterial burden was sharply elevated 72 hours after infection in the absence or presence of 1,25D when macrophages were co-cultured with IL1R1-depleted SAECs. In contrast, co-culture of SAECs transfected with control siRNAs eliminated net mycobacterial growth in the presence of 1,25D (**Figure 6C**), consistent with experiments described above. To determine if epithelial secretion of DEFB4 was responsible for the increased control of mycobacterial proliferation, we transfected SAECs with either siRNA against *IL1R1* or *DEFB4* transcripts 36 hours prior to their co-culture with infected macrophages. Reduced expression of *DEFB4* was verified by qPCR in samples collected 72 hours after the initiation of their co-culture (**Figure 6D**). CFU assays were performed at 72 hours after infection and demonstrated that siRNA-mediated knockdown of *DEFB4* expression permitted levels of bacterial proliferation similar to what was observed in knockdown of *IL1R1* (**Figure 6E**). Taken together, these data reveal that IL-1β secreted from infected macrophages drives a paracrine signaling cascade which contributes to control of mycobacterial burden in our culture system.

**Figure 7:**
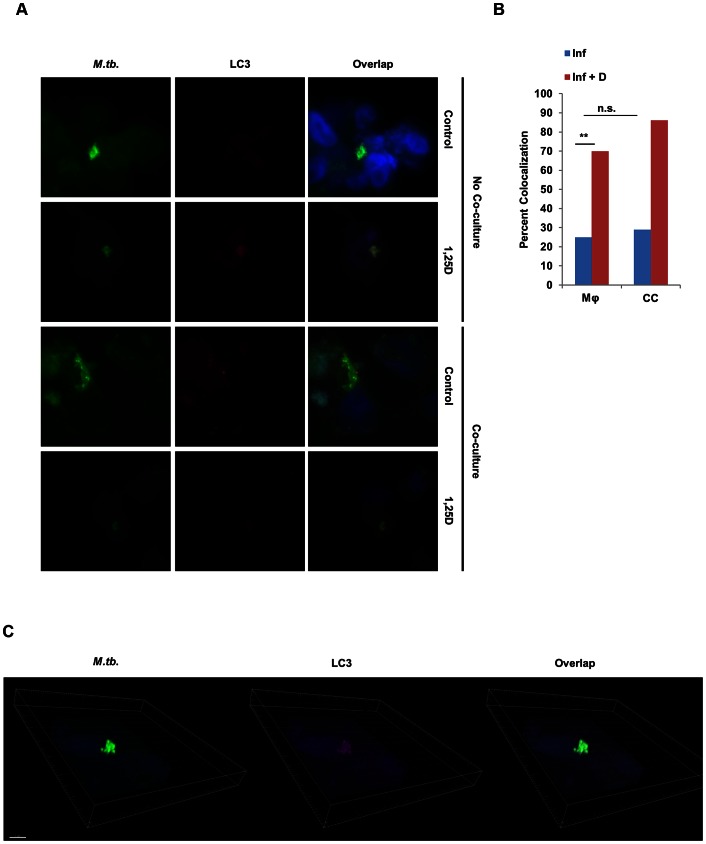
Co-culture with SAECs does not enhance autophagy in *Mtb*-infected macrophages. (**A**) Representative bright-field microscopy of infected macrophages cultured in the absence or presence of 1,25D and/or a transwell containing SAECs. Cells were fixed and probed with an antibody against *Mtb*, LC3, and the nuclear stain DAPI. (**B**) Percent of *Mtb* that colocalized with LC3 signal under each condition as determined by counting populations of infected macrophages across at least 10 confocal images, with at least two infected cells per image. Statistical significance was determined by a two-tailed Fisher's exact test (**P<0.01). (**C**) 3-dimensional rendering of confocal stacks of infected macrophages treated with 1,25D to demonstrate colocalization of LC3 and *Mtb* signal.

**Figure 8:**
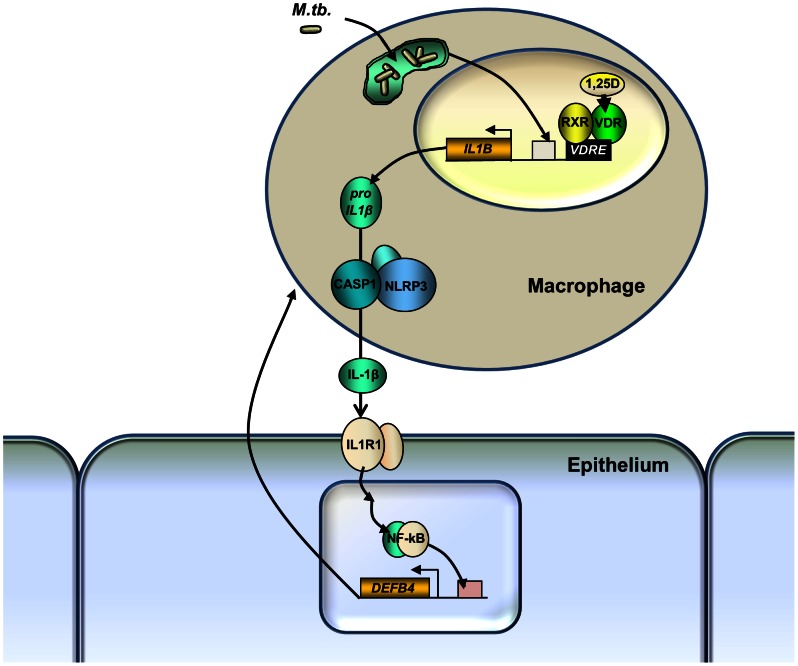
Model of paracrine macrophage-epithelial cell signaling cascade driven by 1,25D and IL-1β. *IL1B* gene expression is driven by infection and 1,25D, and pro-IL-1β maturation is dependent on the NLRP3 inflammasome. Released IL-1β signals via IL1R1 on adjacent epithelial cells to induce expression of *DEFB4*. The release of DEFB4 induced by IL-1β, in combination with 1,25D, leads to control of mycobacterial proliferation in the macrophage.

The contribution of 1,25D to reducing mycobacterial burden in macrophages arises at least in part from its capacity to enhance autophagy in infected macrophages in a CAMP-dependent manner [38], [55], a critical mechanism for control of intracellular pathogens. To understand if epithelial CAMP or DEFB4 secretion was helping to control bacterial burden by enhancing autophagy in macrophages, even if CAMP was undetected in the media supernatant in control experiments, we probed for colocalization of *Mtb* and LC3 protein, a marker of autophagosomes, in (1,25D-treated) infected macrophages 3 days after infection. Using bright-field fluorescence microscopy, we visualized *Mtb* and the presence of any colocalized LC3 (**Figure 7A**). Quantification of the number of times in which mycobacteria were found in LC3-containing membrane structures in confocal imaging revealed that the frequency of colocalization increased when infected macrophages were treated with 1,25D; however, the presence of SAECs in co-culture had no impact on the degree of colocalization, both in the absence and presence of 1,25D (**Figure 7B**). Finally, analysis of 3-dimensional stacks of confocal images taken from 1,25D-treated conditions confirmed that the signal from *Mtb* colocalized with LC3-containing structures (**Figure 7C**). Taken together, these data reveal that the decrease in CFU observed in co-culture experiments, which is dependent on paracrine IL-1β signaling and the reciprocal secretion of DEFB4 from epithelial cells (**Figure 8**), is not a consequence of an increase in autophagy in macrophages.

## Discussion

In this study we have presented the first large scale microarray profile to determine the host macrophage transcriptomic responses to *Mtb* infection and the effect of 1,25D on those responses. Analysis of this data demonstrated that infection markedly changed the profile of genes regulated by 1,25D. ChIP studies also revealed that infection enhances DNA binding by the VDR. These data suggest infection induces large-scale changes in chromatin that modify the availability of VDREs for binding by the VDR throughout the genome. Additionally, it is clear from this work that studies of 1,25D target genes in uninfected macrophages are not an accurate depiction of its contribution to host responses in the face of an active infection. A potential limitation of this study was our initial use of THP-1 cells for microarray analysis of target genes. As this is cell line is derived from a monocytic leukemia, not all of the genes identified as being regulated may be found under similar experimental conditions using primary human alveolar macrophages.

Pathways analysis of genes differentially regulated by 1,25D in infected THP-1 cells revealed that the dominant function of 1,25D in the context of the innate immune response to *Mtb* is the up-regulation of specific components of the broad cytokine response induced by infection. Previous computational analysis identified VDREs proximal to the *IL1B*, *CCL3*, *CCL4*, *CCL8* genes, but not *IL-8* or *TNF-a* [42]. Probing 1,25D modulation of these cytokines *in vivo* is complicated by our results showing that these VDREs are not conserved in mouse, rabbit, or guinea pig (this paper and Verway et al, manuscript in preparation), the model organisms typically used to study *Mtb* infection. Despite the fact that IL-1β signaling also is critical for innate immune responses to *Mtb* in these animal models, it would appear that they would not be appropriate for *in vivo* modeling of the modulatory effects of vitamin D on the early innate immune responses to infection.

1,25D markedly enhanced mRNA levels and secretion of IL-1β in *Mtb*-infected macrophages. Our data reveal that 1,25D is acting primarily at the level of *IL1B* gene transcription without affecting the levels of inflammasome, as substantial levels of pro-IL-1β were seen in uninfected macrophages after 1,25D treatment, whereas secretion required infection. Furthermore, of the cytokines whose secretion was elevated by 1,25D treatment after infection (CCL3, CCL4, CCL8, IL-8, and TNF-α), treatment did not induce their secretion from uninfected cells. This would suggest that 1,25D is acting to prime and potentiate inflammatory innate immune responses, without inducing any unwanted inflammation in a resting state.

IL-1β and IL1R1 are essential for survival in mouse models of *Mtb* infection [8], [9], [10], [11]. IL-1β is also of critical importance during the early stages of infection as shown by *in vitro* infections using macrophages from these knockout mice [11]. Additionally, it has been demonstrated that *Mtb* expresses *zmp1*, a metalloproteinase that prevents phagolysosomal maturation by inhibiting inflammasome-mediated IL-1β cleavage, a mechanism of virulence that suppresses the host response [40]. siRNA-mediated knockdowns of various inflammasome sensors demonstrated that the NLRP3 inflammasome is responsible for IL-1β secretion under all conditions, in agreement with previously published data looking at mycobacterial infection in mouse and human macrophages [22], [23]. While mouse genetic models have revealed that IL-1β signaling plays a key role in *Mtb* resistance, *nlrp3*−/− and *casp1*−/− mice showed no deficiency in the production of IL-1β, control of bacterial burden, or survival [23]. Even though ASC was important in this model, this may be because ASC-null mice are deficient in antigen presentation and DC trafficking due to a loss of Dock2 expression [56]. It is therefore unclear at this time whether the NLRP3 inflammasome is required *in vivo* for control of *Mtb* infection.

Previous studies on the role of IL-1β in the innate immune response to infection have focused on its capacity to control infection by autocrine signaling from infected macrophages. While epithelial cells are known to have important immunologic function in other diseases and contexts [4], [57], [58], they are usually not considered to be an important part of the innate immune response to *Mtb* infection. Primary upper respiratory epithelial cells express DEFB4 in response to IL-1β stimulation *in vitro* [6]. Our data demonstrate that the IL-1β secreted from infected macrophages has the capacity to elicit an antimycobacterial response from small airway epithelial cells. Importantly, siRNA-mediated knockdown of epithelial IL1R1 or DEFB4 expression significantly negated the control of *Mtb* growth in co-cultured macrophages. Modeling of these findings *in vivo* will be necessary to fully understand the extent of the contribution of epithelial cells to the innate immune response in this context. Doing so in primates would pose a major undertaking, and it would be difficult to assess the contribution of the reciprocal signaling cascade between alveolar macrophages and epithelial cells in such a model, as it would likely occur in the acute response to a very low dose exposure.

Macrophage-epithelial cell co-culture controls mycobacterial burden but did not induce autophagy, and epithelial knockdown of IL1R1 expression negated any protective benefit. Taken together, our data suggest that paracrine secretion of DEFB4 from epithelial cells provides a more substantial level of protection against infection than DEFB4 production by macrophages. Previous studies have provided evidence for a reduction in bacterial burden when *Mtb*-infected macrophages are treated with 1,25D *in vitro* [38], [39]. We find that this effect is limited, and that bacterial burden is elevated 3 days after infection with virulent *Mtb*, representing a failure to control the infection. From this study it is clear that more complete models of the innate immune response are required to understand the full effect of 1,25D.

Correlations between vitamin D deficiency and a higher incidence of TB have repeatedly been observed [27], [28], [29]. Clinical trials did not reveal a substantial benefit of vitamin D in the treatment of active disease [59], [60], although a recent study showed that vitamin D supplementation accelerated the resolution of inflammatory responses during treatment for *Mtb* disease [61]. Our results would suggest that the optimal point of treatment would be vitamin D supplementation to bolster innate responses and prevent infection. Such a program would be economically viable considering the negligible cost of vitamin D supplementation as compared to antibiotic chemotherapy, and a useful prophylactic measure against drug-resistant tuberculosis. Our mechanistic data supports the idea that serum levels of vitamin D may be causally important for defense against *Mtb* exposure, but clinical trials would be required to understand this.

## Materials and Methods

### Ethics statement

Intraperitoneal macrophages were acquired from C57BL/6 mice as outlined in an animal use protocol approved by McGill University (Permit #2010-5860) according to Canadian Council on Animal Care guidelines. De-identified human peripheral blood was purchased from Research Blood Components (Boston, MA). Following informed written consent, blood was collected by venipuncture from healthy adult volunteers, recruited by Research Blood Components. Protocols for the collection of whole blood for research purposes were approved by New England Institutional Review Board.

### Tissue culture

THP-1 cells (TIB-202, ATCC) were cultured in RPMI-1640 (Wisent) with 10% FBS. SAEC cells were acquired following informed consent, permission, and ethical approval by Lonza, and were cultured in SAGM (Lonza), as directed by manufacturer. These cells were isolated by a proprietary method from the 1 mm bronchiole of the lung, which includes alveoli. H37Rv was cultured to mid-log phase in a rolling incubator at 37°C in Middlebrook 7H9 (Difco) with .05% Tween-80 and 10% ADC enrichment (BD Biosciences).

### Macrophage infections

1×10^6^ THP-1 cells were terminally differentiated by 2×10^−8^M PMA for 24 hours in RPMI with 10% charcoal stripped FBS, inducing cell cycle arrest. H37Rv cultures were centrifuged and pellets were resuspended in RPMI-1640 with 10% charcoal stripped FBS and .05% Tween-80 and clumping was disrupted by repeated passage through a 27-gauge needle. Media was removed from THP-1 cells and replaced with media containing *Mtb* in the indicated multiplicity of infection (MOI) for 4 hours. THP-1 cells were then vigorously washed three times with RPMI to remove extracellular bacteria, followed by incubation in RPMI with 10% charcoal stripped FBS containing either vehicle (DMSO) or 1,25D at a final concentration of 10^−7^ M.

### RNA extraction and qPCR/microarray

RNA extraction was performed with TRIzol/chloroform (Invitrogen) as per manufacturers' instructions. RT was performed with iScript cDNA Synthesis Kit (Bio-Rad) and qPCR was performed with SsoFast Eva Green with low ROX (Bio-Rad) on an Eco qPCR cycler (Illumina), normalizing expression to β-actin and 18S. Primers used are listed in Table S4. Human Gene 1.0 ST arrays (Affymetrix) were used to measure samples from two independent experiments, each performed in triplicate. Microarray data presented is from one triplicate set, which is representative of the other.

### Western blots and immunoprecipitation

Protein extracts from THP-1 cells were prepared in lysis buffer (10 mM Tris, pH 7.5, 150 mM NaCl, 1% Triton X-100, 1 mM phenylmethylsulfonyl fluoride, 0.2 mM sodium orthovanadate, 0.5% Nonidet P-40) and processed for Western blotting and separated on Tris-HEPES-SDS gradient protein gels (Pierce) using standard transfer and blotting protocols. Immunoprecipitation was performed as described in [62], using 2 µg of either rabbit serum IgG (sc-2027) or α-caspase-1 p10 (sc-515, Santa Cruz) antibody for each sample.

### Reagents, ELISAs, and Western blot antibodies

Antibodies: α-IL-1β (MAB601, R&D Systems), α-AIM2 (ab93015, abcam), α-caspase-1 p20 (sc-1780, Santa Cruz), α-actin (sc-1616, Santa Cruz), α-VDR (sc-1008, Santa Cruz), α-ASC (sc-271054, Santa Cruz), murine serum IgG (Sigma). Recombinant IL-1β was purchased from Millipore. ELISAs for DEFB4 (Abnova) and LL-37 (Hycult Biotech) were performed in accordance with the manufacturer's instructions.

### Macrophage CFU assay

At the indicated time points, tissue culture plates were centrifuged to pellet any liberated mycobacteria and non-adherent cells. Media was aspirated and macrophages were lysed with water for 5 min, after which an equal volume of 2x 7H9 with .1% Tween-80 was added. Samples were vigorously resuspended and plated in serial dilution on Middlebrook 7H10 (Difco) with 10% OADC enrichment (BD Biosciences).

### Human macrophage isolation

Following informed consent, blood was drawn from healthy adults. Buffy coats were isolated from whole blood by Ficoll density-gradient centrifugation using Ficoll-Paque Premium (GE Healthcare), and thrice resuspended in PBS and centrifuged to remove platelets. Monocytes were purified from the total leukocyte population by sorting of adherent cells after 2 hours, by washing the culture plates twice with RPMI-1640 with 20% human serum. Cells were allowed to differentiate into naïve macrophages over 6 days in RPMI-1640 with 20% human serum before infection, with media changes every two days throughout.

### Mouse macrophage isolation

Intraperitoneal macrophages were elicited from C57BL/6 mice (Jackson) by intraperitoneal injection of 2 ml of 3% thioglycolate solution. Three days later, macrophages were collected by lavage of the peritoneal cavity. Macrophages were purified using CD11b Microbeads (Miltenyi Biotech) and allowed to adhere for 24 hours before infection.

### siRNA-mediated knockdowns

One day after PMA-induced differentiation, siRNAs targeting *CASP1*, *AIM2*, *IL1B*, *NLRP3*, *NOD2*, and the non-targeting scrambled control (NC1) (Integrated DNA Technologies) were transfected into THP-1 cells using Transductin (Integrated DNA Technologies) in 10% Q-serum, according to manufacturer's instructions. After 4 hours, media was replaced with RPMI with 10% charcoal stripped FBS. After another 48 hours, cells were infected with H37Rv in accordance with the above protocol.

### THP-1 to SAEC media transplant

THP-1 cells were not infected or infected with H37Rv followed by treatment with DMSO or 1,25D at a final concentration of 10^−7^ M for 24 hours. Media was filter sterilized by passage through 0.20 µm filters. Media was then incubated with anti-IL-1β or non-specific murine IgG for 30 minutes before transfer to epithelial cell cultures. Cultures were harvested for RNA after 24 hours.

### Transwell co-culture

Epithelial cells were seeded in transwell buckets with 0.4 µm pores (Corning) and cultured in RPMI with 10% charcoal stripped FBS 48 hours before infection of the THP-1 cells. After the THP-1 cells were infected for 4 hours at a MOI of 5 and washed, SAECs were placed in co-culture with H37Rv-infected THP-1 cells by transferring the buckets containing the epithelial cells to the plates containing the infected macrophages, and then treated with DMSO or 1,25D (10^−7^ M). At indicated time points, plates were centrifuged and both cell populations were lysed with water and pooled for CFU assay, performed as described above.

### Immunofluorescence and image acquisition

Following 4% paraformaldehyde fixation over 10 minutes, cells on coverslips were washed with 100 mM glycine and then PBS. Cells were then permeabilized with 0.1% Triton X-100 for 5 minutes. Cells were incubated in PBS-0.2% BSA during 5 minutes. Primary antibody against LC3 (Novus Biological, dilution 1/300) was incubated with coverslips for 1 hour at 37°C in a humidified chamber in phosphate-buffered saline with 1% BSA. Following 3 washes, cells were incubated with the secondary antibody anti-rabbit Alexa-647 (Life Technologies, dilution 1/1000) and with the anti-*Mtb* antibody coupled to FITC (Abcam ab20962, dilution 1/100) for 45 minutes at RT in the dark. Slides were mounted in Vectashield containing DAPI (Vector) and observed with a Zeiss Axiovert X100 bright field microscope or a Zeiss LSM510 X100 confocal microscope. Images were acquired with LSM510 software. Stacks of confocal images, 3D reconstitution, and quantification of percent of colocalization were performed with Imaris 7.4 and the figures were assembled with Photoshop (Adobe).

### Caspase-1 activity assay

Total caspase-1 activity was assayed using ICE/Caspase-1 Colorimetric Protease Assay Kit (Millipore) as per manufacturers' instructions. Cell lysates were incubated with YVAD-pNA and read in a spectrophotometer at 405 nM.

### Secreted cytokine and chemokine quantification

Media from THP-1 cells was collected 24 hours after treatment and sterilized by passage through .20 µm filters. Levels of secreted cytokines and chemokines were assayed by Milliplex Human Cytokine Panels 1, 2 and 3 (Millipore) and read on a BioPlex (Bio-Rad).

### ChIP assays

Infected and uninfected THP-1 cells were collected after 24 hours of treatment. Cells were fixed for 20 minutes with 1% formaldehyde, and washed with 1.25 µM glycine. Following cell and nuclei lysis, chromatin was sonicated for 75 cycles of 10 s ON/20 s OFF on a Bioruptor Sonicator (Diagenode). IPs were then performed with either normal rabbit IgG, 6 µg of anti-VDR (Santa Cruz sc13133) or 6 µg of anti-RNA Polymerase II (Abcam anti-polIICTD #ab5131). Primers used for region amplification are listed in Table S4. Quantification of immunoprecipitated material was performed by qPCR and normalized for input DNA.

### Statistical analysis

Student's t-test, ANOVA, or a two-tailed Fisher's exact test was performed where indicated using GraphPad software. For microarray samples, Flexarray v1.6 software was used to normalize overall chip signal using the Affymetrix Power Tools (APT) Robust Multi-Array Average (RMA) algorithm. The EB (Wright and Simon) algorithm was used for statistical analysis to calculate fold transcript expression and significance between of each experimental condition relative to the uninfected, untreated condition (NI).

### LDH assay

Cell-free media supernatant was collected from infected macrophages with and without SAECs in co-culture. Cytotoxicity Detection Kit (LDH) from Roche was used in accordance with manufacturer's instructions.

### List of genes and proteins and their corresponding RefSeq IDs

IL-1β: NM_000576/NP_000567, Caspase-1: NM_001223/NP_001214, NLRP3: NM_001079821/NP_001073289, AIM2: NM_004833/NP_004824, NOD2: NM_022162/NP_071445, DEFB4: NM_004942/NP_004933, CATH: NM_004345/NP_004336

## Supporting Information

Figure S1 **Sequence and position of the** ***IL1B*** **VDRE in the human genome relative to the** ***IL1B*** **transcription start site.** Each half site of the VDRE is indicated by the nucleotides which are capitalized.
(TIF)

Figure S2 **VDR protein expression in Mtb-infected macrophages.** Western analysis of VDR expression in THP-1 cells that were not infected (NI) or H37Rv-infected (I) and treated with vehicle or 100 nM 1,25D (+D) for 24 hours. Data are from one experiment and representative of two.
(TIF)

Figure S3 **Evolutionary conservation of the** ***IL1B*** **VDRE in mammals.** Comparison of the *IL1B* VDRE loci (4287 bases upstream of the human *IL1B* transcription start site) across various mammalian species, as aligned by the UCSC Genome Browser. Agreement with the human sequence is indicated by the nucleotide base appearing in red. Both VDRE half-sites are indicated by the position of the black boxes.
(TIF)

Figure S4 **Regulation of** ***il1b*** **gene expression by 1,25D in primary cultures of mouse macrophages (Mφ) measured after 24 hours.** Regulation of the VDR target gene cyp24 is provided as a positive control for 1,25D signaling. Values are normalized to untreated control for each time point. All data are from one experiment and representative of two independent experiments (n = 3, mean, s.d.). **P<0.01 as determined by Student's t-test relative to untreated.
(TIF)

Figure S5 **Caspase-1 expression and activity in control and infected THP-1 cells.** (**A**) Expression of caspase-1 analyzed by western blotting in THP-1 cells that were not infected (NI) or H37Rv-infected (I) and treated with vehicle or 100 nM 1,25D (+D) for 24 hours. Data is from one experiment and representative of at least five. (**B**) Enzymatic activity of caspase-1 in cell lysates as measured by cleavage of YVAD-pNA substrate. Data are from four experiments (mean and s.e.m.). Indicated p-values were calculated using Student's t-test relative to respective uninfected controls. (**C**) Western blot of ASC in cell lysates where caspase-1 p10 antibody was used for immunoprecipitation. Protein lysates from infected THP-1 cells were collected 24 hours after infection, and data are representative of two independent experiments.
(TIF)

Figure S6 **Control experiments for effects of knockdown of pattern recognition receptor expression on IL-1β secretion from infected THP-1 cells.** (**A**) siRNA-mediated knockdown of *AIM2* expression in uninfected and infected THP-1 cells as quantified by RT/qPCR. (**B**) qPCR quantification of NOD2 and NLRP3 gene expression in *Mtb*-infected THP-1 cells after transfection with control or specific siRNAs. Values are expressed as fold relative to control siRNA. All data are from one experiment and representative of at least three independent experiments (n = 3, mean, s.d.).
(TIF)

Table S1 **Microarray results and statistical analysis from NI+D, I, and I+D conditions measured relative to NI. The THP-1 cells were either not infected (NI) or H37Rv-infected (I) and treated with vehicle (DMSO) or 100 nM 1,25D (D) for 24 hours before RNA extraction.** Samples were prepared in triplicate and run on Human Gene 1.0ST microarrays from Affymetrix. Overall chip signal was normalized using RMA. Fold expression and significance relative to NI was calculated using EB (Wright and Simon).
(XLSX)

Table S2 **Number of genes detected by microarray that were up- or down-regulated at least 5-fold relative to the uninfected, vehicle-treated control.** THP-1 cells were not infected (NI) or infected with H37Rv (I) and treated with vehicle or 100 nM 1,25D (D) for 24 hours before RNA isolation.
(TIF)

Table S3 **Secretion profile of media samples taken from cells used for microarrays.** THP-1 cells were either not infected (NI) or infected with H37Rv (I) and treated with vehicle or 100 nM 1,25D (+D) for 24 hours. All units are in pg/ml, and empty boxes represent no detectable increase above background. Data are from three experiments (mean and s.e.m.). The following were not detected: IFNγ, IL-2, IL-3, IL-5, IL-10, IL-15, IL-17, INFβ, IL-11, IL-29, XCL1, CXCL5, CXCL6, CXCL7, CCL14a.
(TIF)

Table S4 **Primer sets used for ChIP and qPCR assays.**
(TIF)
