# Supplementary figures and images for: Vitamin D Induces Interleukin-1β Expression: Paracrine Macrophage Epithelial Signaling Controls M. tuberculosis Infection
Source: PLoS Pathog. 2013 Jun 6;9(6):e1003407. doi: 10.1371/journal.ppat.1003407 (PMC3675149; doi:10.1371/journal.ppat.1003407)

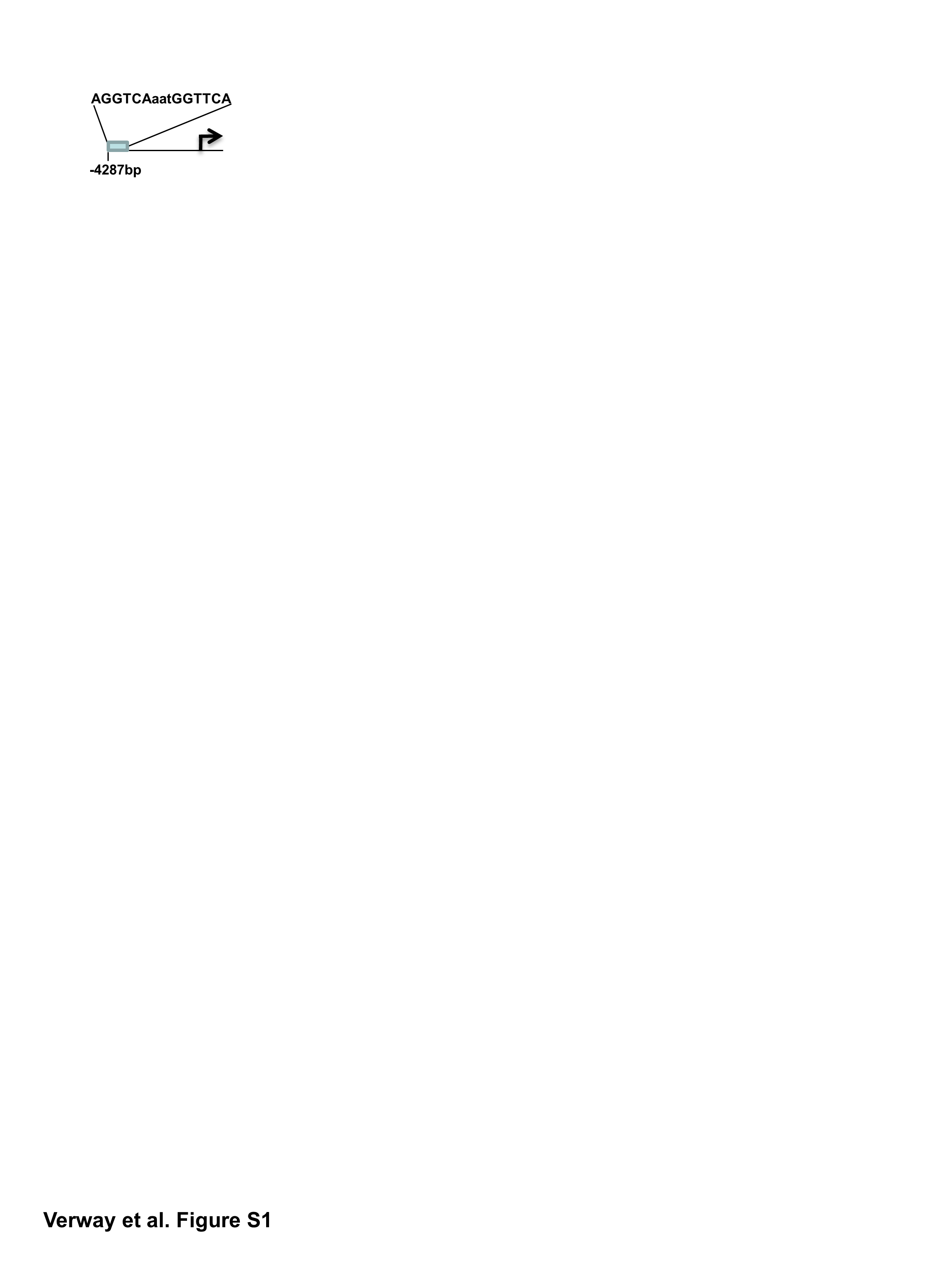

Supplement: Figure S1 — Sequence and position of the IL1B VDRE in the human genome relative to the IL1B transcription start site. Each half site of the VDRE is indicated by the nucleotides which are capitalized. (TIF) [file ppat.1003407.s001.tif]

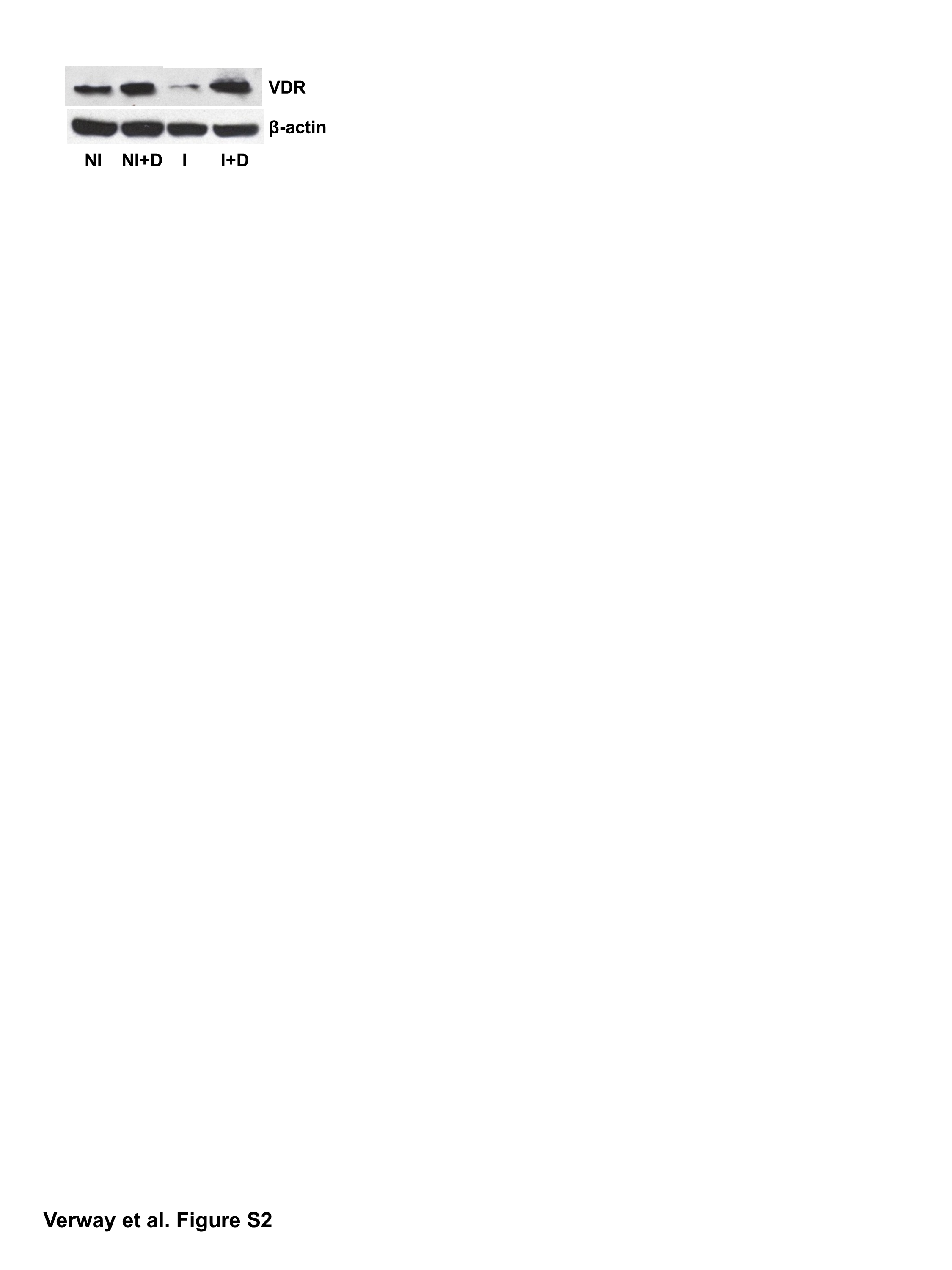

Supplement: Figure S2 — VDR protein expression in Mtb-infected macrophages. Western analysis of VDR expression in THP-1 cells that were not infected (NI) or H37Rv-infected (I) and treated with vehicle or 100 nM 1,25D (+D) for 24 hours. Data are from one experiment and representative of two. (TIF) [file ppat.1003407.s002.tif]

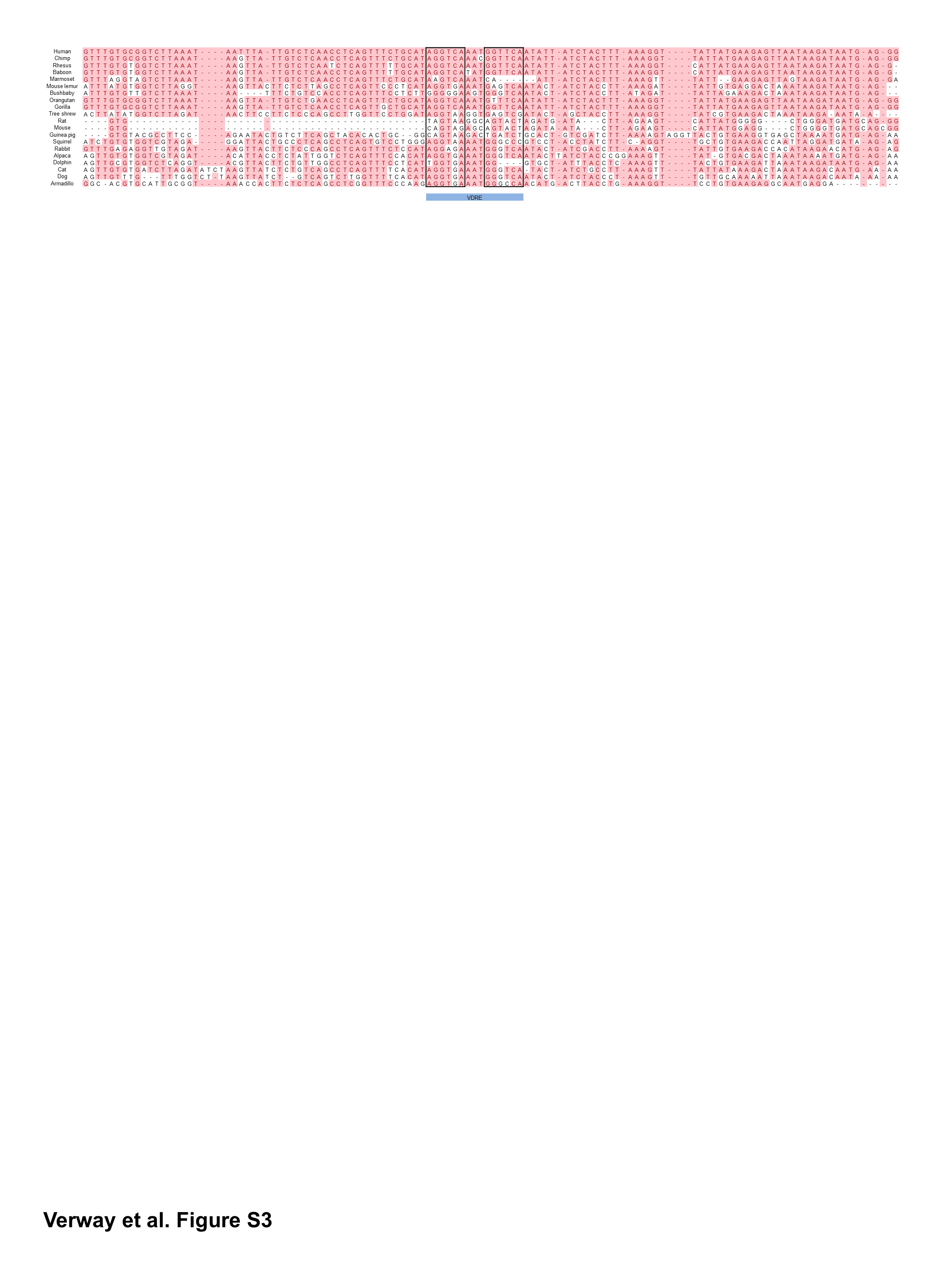

Supplement: Figure S3 — Evolutionary conservation of the IL1B VDRE in mammals. Comparison of the IL1B VDRE loci (4287 bases upstream of the human IL1B transcription start site) across various mammalian species, as aligned by the UCSC Genome Browser. Agreement with the human sequence is indicated by the nucleotide base appearing in red. Both VDRE half-sites are indicated by the position of the black boxes. (TIF) [file ppat.1003407.s003.tif]

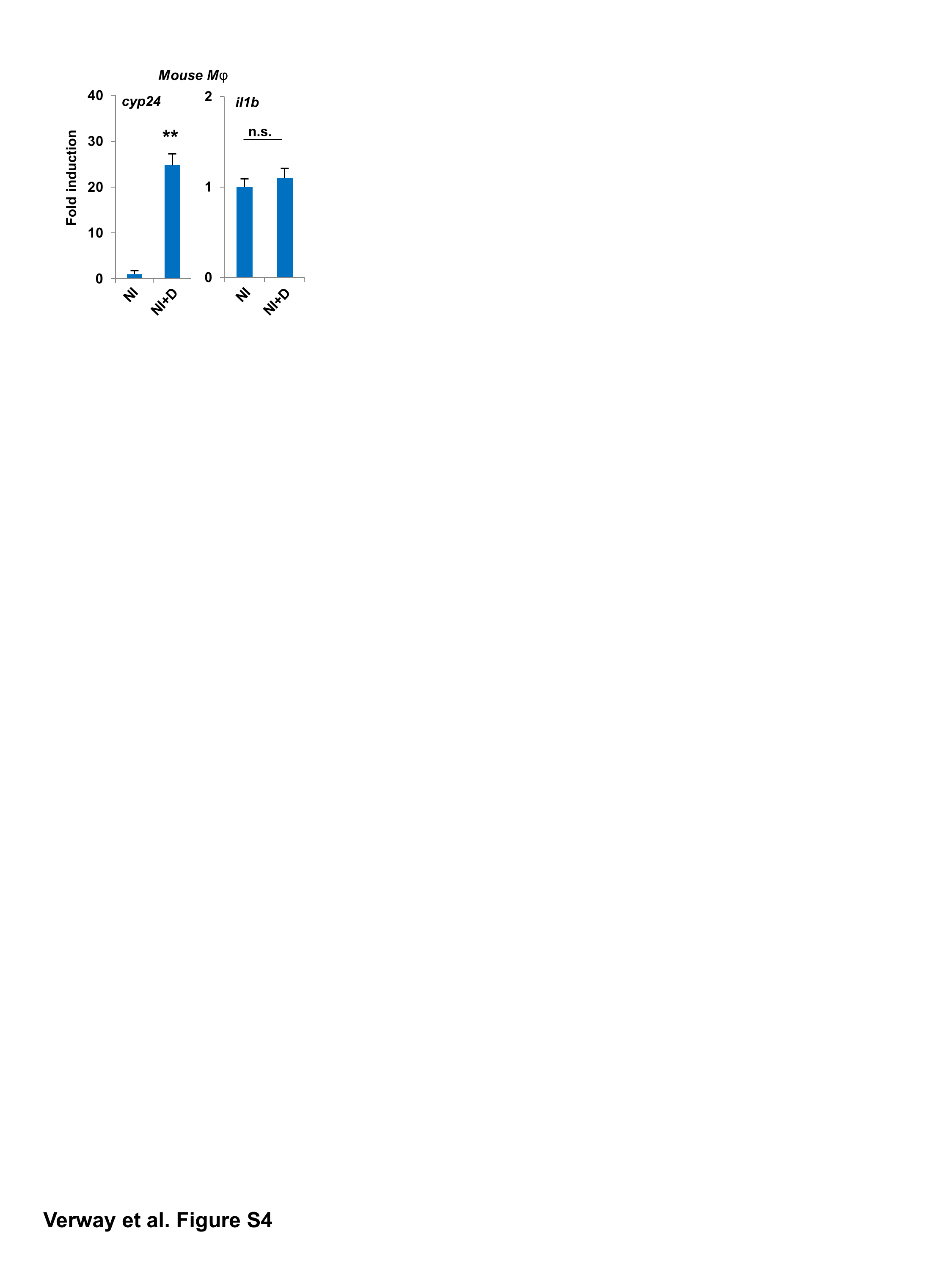

Supplement: Figure S4 — Regulation of il1b gene expression by 1,25D in primary cultures of mouse macrophages (Mφ) measured after 24 hours. Regulation of the VDR target gene cyp24 is provided as a positive control for 1,25D signaling. Values are normalized to untreated control for each time point. All data are from one experiment and representative of two independent experiments (n = 3, mean, s.d.). **P<0.01 as determined by Student's t-test relative to untreated. (TIF) [file ppat.1003407.s004.tif]

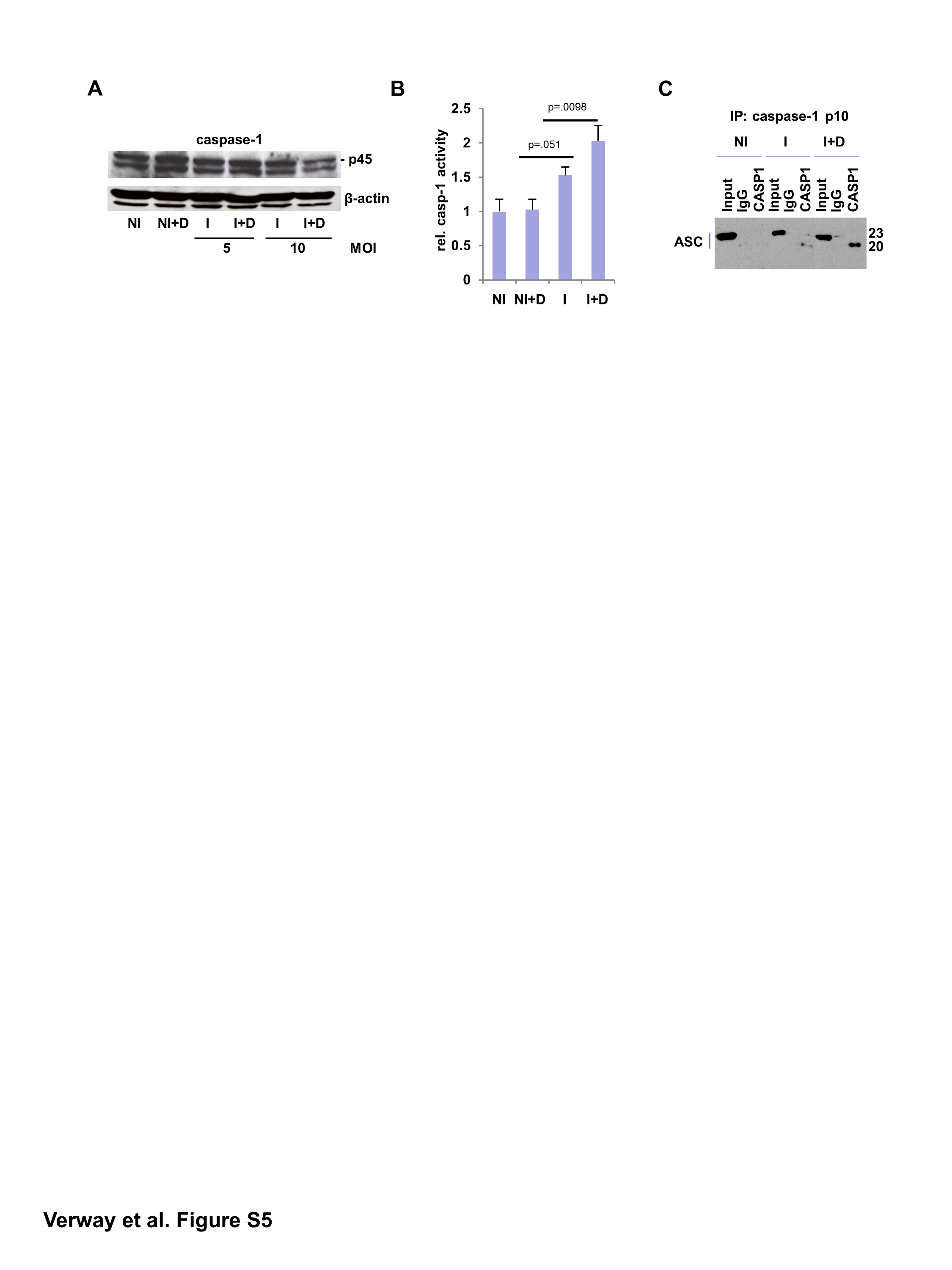

Supplement: Figure S5 — Caspase-1 expression and activity in control and infected THP-1 cells. (A) Expression of caspase-1 analyzed by western blotting in THP-1 cells that were not infected (NI) or H37Rv-infected (I) and treated with vehicle or 100 nM 1,25D (+D) for 24 hours. Data is from one experiment and representative of at least five. (B) Enzymatic activity of caspase-1 in cell lysates as measured by cleavage of YVAD-pNA substrate. Data are from four experiments (mean and s.e.m.). Indicated p-values were calculated using Student's t-test relative to respective uninfected controls. (C) Western blot of ASC in cell lysates where caspase-1 p10 antibody was used for immunoprecipitation. Protein lysates from infected THP-1 cells were collected 24 hours after infection, and data are representative of two independent experiments. (TIF) [file ppat.1003407.s005.tif]

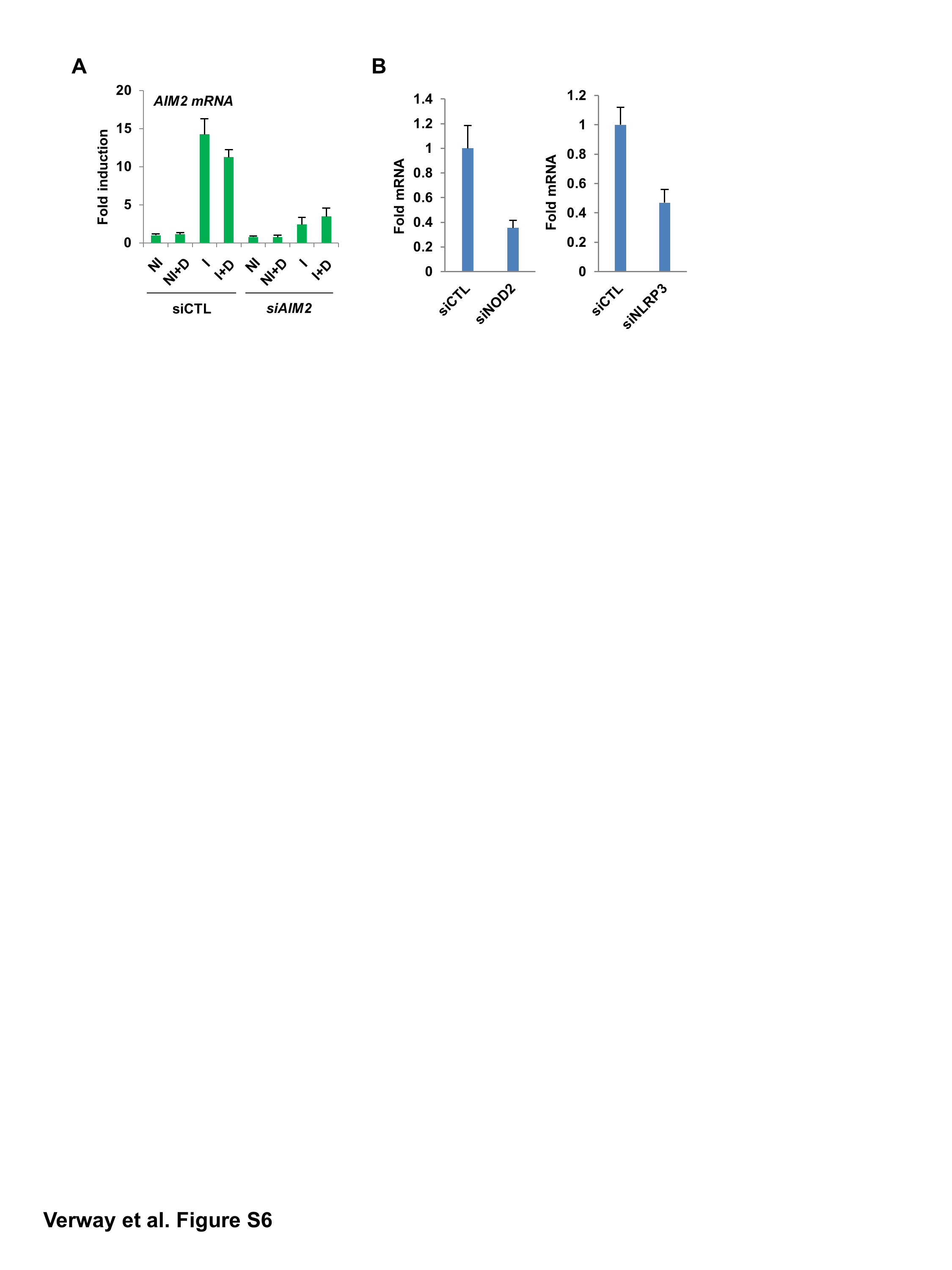

Supplement: Figure S6 — Control experiments for effects of knockdown of pattern recognition receptor expression on IL-1β secretion from infected THP-1 cells. (A) siRNA-mediated knockdown of AIM2 expression in uninfected and infected THP-1 cells as quantified by RT/qPCR. (B) qPCR quantification of NOD2 and NLRP3 gene expression in Mtb-infected THP-1 cells after transfection with control or specific siRNAs. Values are expressed as fold relative to control siRNA. All data are from one experiment and representative of at least three independent experiments (n = 3, mean, s.d.). (TIF) [file ppat.1003407.s006.tif]

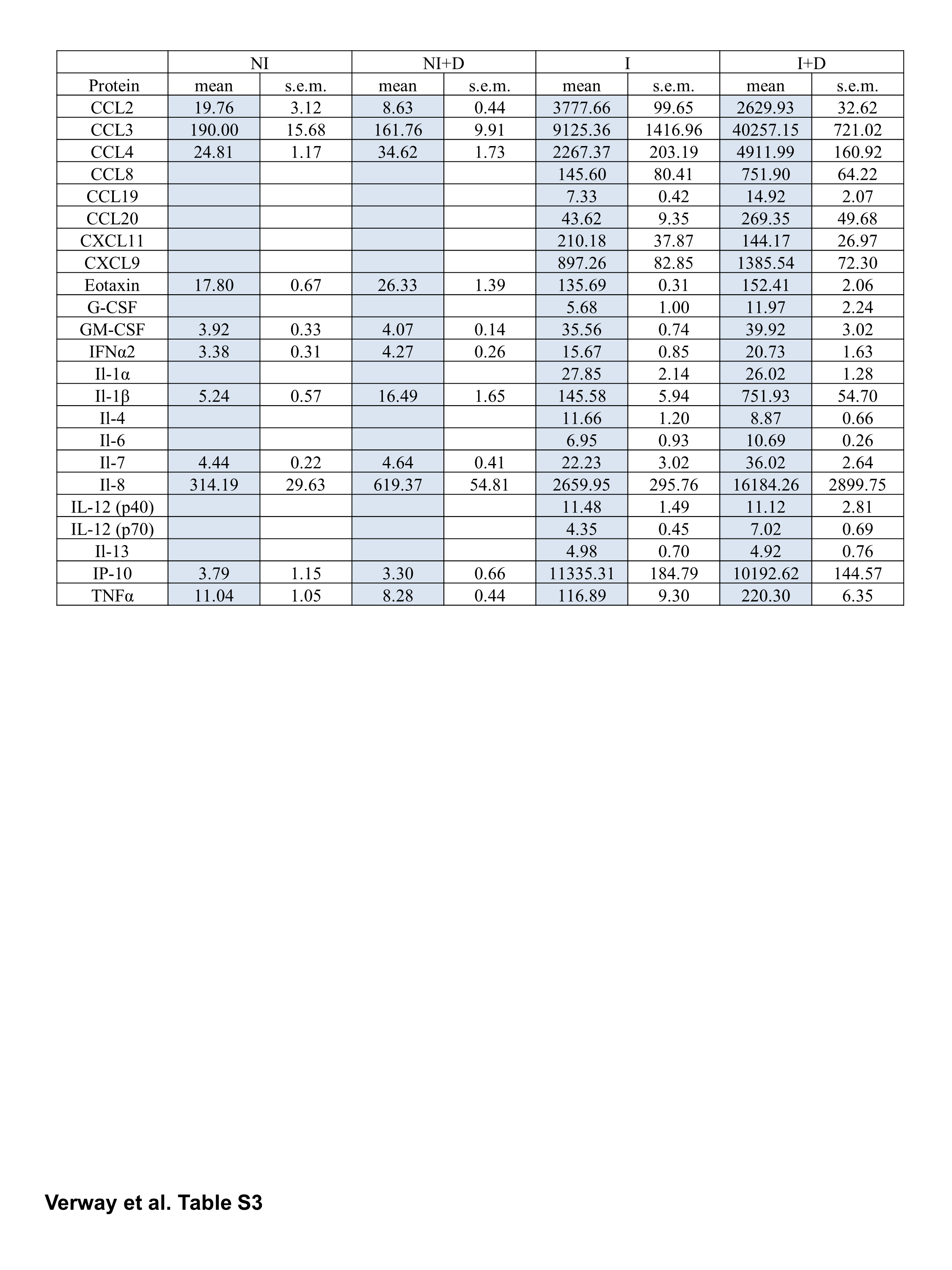

Supplement: Table S3 — Secretion profile of media samples taken from cells used for microarrays. THP-1 cells were either not infected (NI) or infected with H37Rv (I) and treated with vehicle or 100 nM 1,25D (+D) for 24 hours. All units are in pg/ml, and empty boxes represent no detectable increase above background. Data are from three experiments (mean and s.e.m.). The following were not detected: IFNγ, IL-2, IL-3, IL-5, IL-10, IL-15, IL-17, INFβ, IL-11, IL-29, XCL1, CXCL5, CXCL6, CXCL7, CCL14a. (TIF) [file ppat.1003407.s009.tif]
